# Genotype Distribution and Molecular Epidemiology of Hepatitis C Virus in Hubei, Central China

**DOI:** 10.1371/journal.pone.0137059

**Published:** 2015-09-01

**Authors:** Jing Peng, Yanjun Lu, Weiyong Liu, Yaowu Zhu, Xiaoling Yan, Jingxin Xu, Xiong Wang, Yue Wang, Wei Liu, Ziyong Sun

**Affiliations:** 1 Department of Clinical Laboratory, Tongji Hospital, Tongji Medical College, Huazhong University of Science and Technology, Wuhan, China; 2 Department of Public Health, Tongji Hospital, Tongji Medical College, Huazhong University of Science and Technology, Wuhan, China; Centers for Disease Control and Prevention, UNITED STATES

## Abstract

**Background:**

Little is known about the molecular epidemiology of hepatitis C virus (HCV) infection in Central China.

**Methodology/Principal Findings:**

A total of 570 patients from Hubei Province in central China were enrolled. These patients were tested positive for HCV antibody prior to blood transfusion. Among them, 177 were characterized by partial NS5B and/or Core-E1 sequences and classified into five subtypes: 1b, 83.0% (147/177); 2a, 13.0% (23/177); 3b, 2.3% (4/177); 6a, 1.1% (2/177); 3a, 0.6% (1/177). Analysis of genotype-associated risk factors revealed that paid blood donation and transfusion before 1997 were strongly associated with subtypes 1b and 2a, while some subtype 2a cases were also found in individuals with high risk sexual behaviors; subtypes 3b, 6a, and 3a were detected only in intravenous drug users. Phylogeographic analyses based on the coalescent datasets demonstrated that 1b, 2a, 3b, and 6a were locally epidemic in Hubei Province. Among them, subtype 1b Hubei strains may have served as the origins of this subtype in China, and 2a and 3b Hubei strains may have descended from the northwest and southwest of China, respectively, while 6a Hubei strains may have been imported from the central south and southwest.

**Conclusion/Significance:**

The results suggest that the migration patterns of HCV in Hubei are complex and variable among different subtypes. Implementation of mandatory HCV screening before donation has significantly decreased the incidence of transfusion-associated HCV infection since 1997. More attention should be paid to intravenous drug use and unsafe sexual contact, which may have become new risk factors for HCV infection in Hubei Province.

## Introduction

Hepatitis C Virus (HCV) infection is a worldwide health problem. More than 115 million people worldwide are currently infected with HCV, representing a serious cause of chronic liver disease that may progress to cirrhosis and hepatocellular carcinoma [1]. Characterized by its high genetic variability, HCV has been classified into seven genotypes and a large number of subtypes [2]. Different genotypes show distinct geographic distribution patterns, which reflect the differences in epidemiology, including transmission modes and ethnic variability in different countries. To date, HCV genotypes 1, 2, and 3 are universally distributed, causing the majority of cases in the world, while other genotypes are limited to more specific geographical areas. Genotype 4, for example, is mostly found in the Middle East and North Africa; while genotype 5 is common in South Africa [3]. However, such patterns are constantly evolving as a result of changes in transmission modes and other influencing factors such as immigration and global travel.

China, a major Asian country, has approximately 25–50 million HCV-infected individuals, accounting for 1.8–3.7% of the overall Chinese population and approximately 15–30% of the total HCV-infected population worldwide [4]. Two recent studies performed on Chinese people at a nationwide level have demonstrated that there are four major HCV genotypes (genotypes 1, 2, 3, and 6) prevalent in China. Among them, subtype 1b is most prevalent nationwide, followed by genotypes 2, 3 and 6, with substantial regional variation [5, 6]. In general, the distribution pattern of HCV genotypes is relatively simple in the north, with 1b and 2a being the main circulating subtypes. In contrast, the genotypic distribution pattern in the south is more complex, with subtype 1b most prevalent, and 2a, 3a, 3b, and 6a each accounting for a certain proportion [5–12]. However, little is known about the distribution pattern of HCV genotypes in Hubei Province, which is located in central China and has been serving as a major transportation thoroughfare, and therefore may be a potential transmission hub for HCV infection. So far, most studies have mainly focused on molecular epidemiology of HCV infection in limited populations in Hubei, such as intravenous drug users (IDUs) [13], HIV patients [14] and blood donors [5]. The true situation of HCV infection in Hubei is unknown, nor its relationship with those in other regions of China.

The aim of this study was to determine the genotype distribution of HCV and related risk factors, as well as the relationship between genotypes found in Hubei and those from other regions of China, thereby helping in the development of proper public health policies and therapeutic strategies.

## Materials and Methods

### Study participants

**Fig 1:**
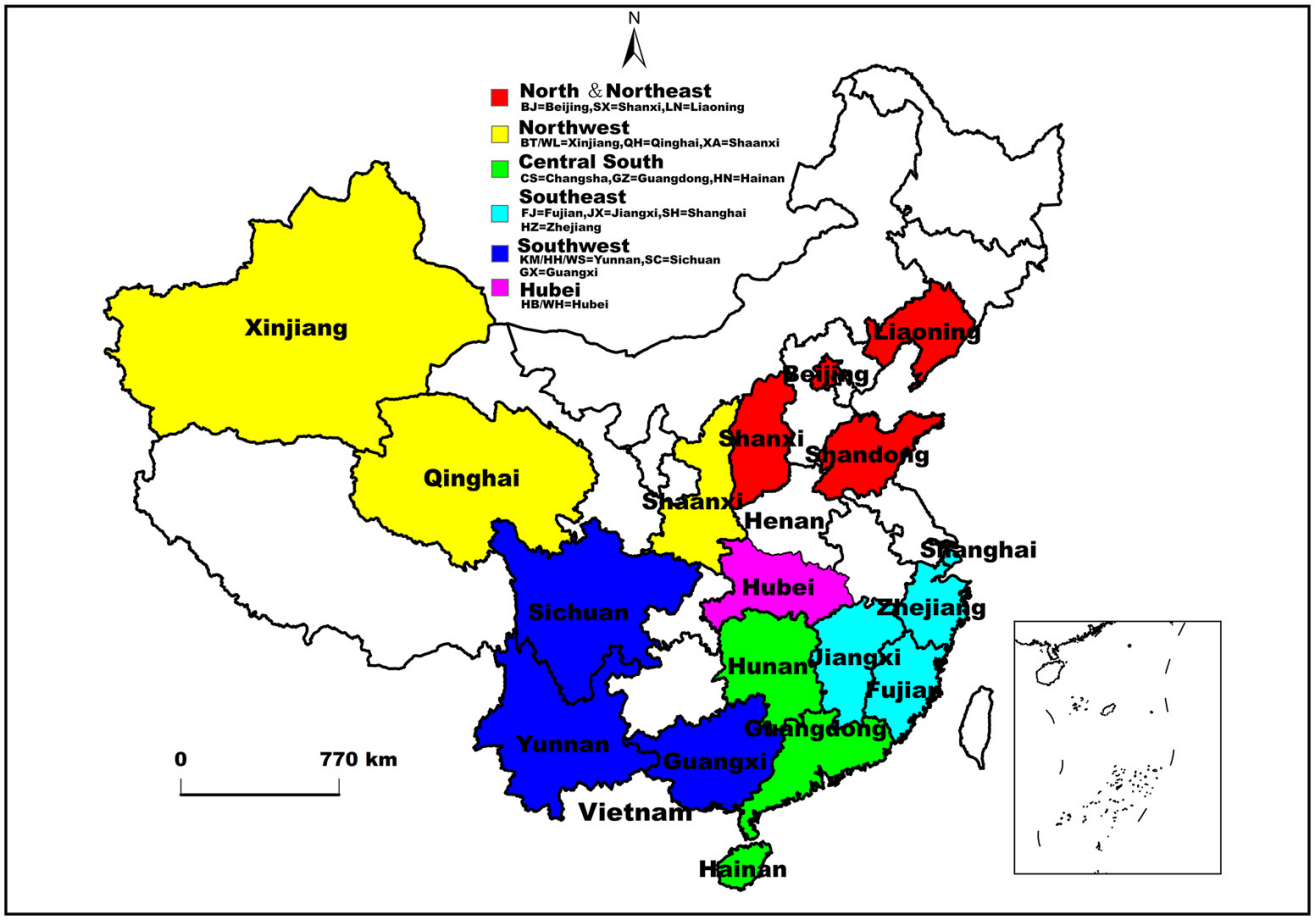
Map highlighting Hubei Province and the 17 provinces and municipalities in China as well as Vietnam where HCV reference sequences were selected. For easier distinction and comparison, the 16 provinces and municipalities other than Hubei Province were divided into five larger regions according to a previous nation-wide study [5] with slight modification: (i) the north-northeast (Beijing, Shanxi, and Liaoning), (ii) the northwest (Xinjiang, Qinghai, and Shaanxi), (iii) the central south (Hunan, Guangdong, and Hainan), (iv) the southeast (Fujian, Jiangxi, Zhejiang, and Shanghai), (v) the southwest (Sichuan, Yunnan, and Guangxi). Accordingly, five colors, red, yellow, green, cyan, and blue were used to mark these five regions on the map. For Hubei, magenta was used, and this color scheme is indicated above the map.

Participants were recruited from Tongji Hospital of Tongji Medical College of Huazhong University of Science and Technology in Hubei Province, central China (Fig 1), from July 2013 to December 2014. These patients were determined positive for HCV-antibodies (anti-HCV) when admitted to various departments and tested for a panel of pathogens, i.e., hepatitis B virus (HBV), HCV, treponema pallidum (TP), and human immunodeficiency virus (HIV) prior to transfusion. Written consents were obtained and the participants were interviewed by doctors and trained research staff to determine their demographics and risk factors associated with HCV infection. Blood samples were centrifuged and the supernatants were stored at -70°C for HCV genotyping. Patients were informed that the tests requested in the study were designed for epidemiological research and would be used to determine the dimension and characteristics of HCV infection in Hubei. Ethical approval, covering the study protocol, was obtained from the Human Ethics Committee of Tongji Medical College.

### Serological assays

Detection of serum hepatitis B surface antigen (HBsAg) and anti-HCV, as well as antibodies to HIV and TP (anti-HIV and anti-TP) was performed at the Department of Clinical Laboratory of Tongji Hospital with corresponding Architect reagents (Architect i2000, Abbott Diagnostics, Abbott Park, IL). Detection of HCV RNA in plasma was performed with a commercially available QIAGEN HCV kit (careHCV PT-PCR Assay V2; QIAGEN Diagnostics, Shenzhen, China). All tests were carried out in accordance with the manufacturers’ instructions.

### HCV RNA extraction, RT-PCR amplification, and sequencing

HCV RNA was extracted from 200 ml of plasma by using High Pure Viral Nucleic Acid Kit (Roche Diagnostics, Indianapolis, IN, USA) according to the recommended protocol. cDNA was synthesized from 15.5 μl extracted RNA with M-MLV Reverse Transcriptase (Promega Corporation, Madison, WI, USA) and random hexaprimers. The primers of the partial NS5B region (H77 positions: 8332–8673 nt) for semi-nested PCR were described elsewhere [15] with slight modification by Tan et al. [8]: outer forward (OF) 5’- TAT GAC ACC MGY TGC TTT GAY TC -3’, outer reverse (OR) 5’- TTG GAG GAG CAD GAT GTT ATS AGC TC -3’, inner reverse (IR) 5’- GAR TAC CTG GTC ATA GCC TCC GT -3’. First round PCR using primers OF and OR was conducted with the following conditions: 94°C for 2 min, 40 cycles of 94°C for 30 sec, 50°C for 20 sec and 72°C for 40 sec, then a final cycle at 72°C for 7min. Second round PCR using primers OF and IR was conducted with the same condition except that the annealing temperature was 51°C for 20 sec. The forward primer OF was used to sequence the PCR products. Additionally, the partial Core-E1 region (H77 positions: 834–1315 nt) was also amplified and sequenced in order to investigate the incidence of mixed infection and viral recombination events in the present cohort. The primers of the Core-E1 region for semi-nested PCR were described elsewhere [16]: outer forward (OF) 5’- GCA ACA GGG AAY YTD CCY GGT TGC TC -3’, inner forward (IF) 5’- AAY YTD CCC GGT TGC TCY TTY TCT AT -3’, outer reverse (OR) 5’- TTC ATC ATC ATR TCC CAN GCC AT -3’. The PCR conditions applied were the same as those used for amplification of the NS5B region. The OR primer was used to sequence the PCR products of the Core-E1 region. Inconsistent results generated by semi-nested PCR when using NS5B and Core-E1 as the target sequences were confirmed by characterizing the more conserved Core sequences. The PCR was performed as described previously [17].

### HCV genotyping and phylogenetic analyses

After determined, sequences were aligned using CLUSTAL_X (version 2) and then edited by BioEdit (version 7.2.5). HCV genotypes were determined after alignment with reference sequences from the GenBank (available at: http://www.ncbi.nlm.nih.gov/genbank/) followed by co-analyses with 5 reference sequences representing 5 subtypes that were detected in this study. These references were selected according to the recommendations in a recent paper by Smith et al. [2]. Prior to phylogenetic tree construction, the best-fitting substitution model was tested using the jModeltest program (version 2.1.7) on the basis of the Akaike Information Criterion [18], which demonstrated that GTR+I+Г6 (GTR + invariant sites + gamma rate heterogeneity) was the best model for all of the sequence datasets. Under this model, Maximum likelihood (ML) trees were heuristically searched using the SPR (Subtree Pruning and Regrafting) and NNI (Nearest Neighbor Interchange) algorithms implemented in PhyML (version 3.1), with which bootstrap analyses were performed in 500 replicates [19]. With the tree files generated and applied to the Figtree program (version1.4.2) [20], tree topology was displayed and converted into a circular form.

### Phylogeographic analysis

Phylogeographic trees were reconstructed using the Bayesian phylogeographic inference framework implemented in the BEAST software (version 2.2.1) [21, 22], where a Bayesian discrete phylogeographic approach and a Bayesian Stochastic Search Variable Selection (BSSVS) procedure were used to estimate the ancestral locations of the virus and infer the most significant epidemiological links, respectively. Before constructing the trees, site models, demographic models, clock models, and evolutionary rates were estimated using the BEAST package [23, 24], which involved all sequences obtained in this study as well as a large number of reference sequences. These samples were collected during a time span of 7–15 years. Briefly, the combination of the GTR+I+Г6 substitution model, the Bayesian skyline coalescent model, and the uncorrelated exponential clock mode was selected since this combination outperformed other combinations, showing log Bayes factors (BF) ranged between 24.66 and 178.52, which were determined by the Tracer software (version 1.6). With the aforementioned datasets and the BEAST settings described previously [23, 24], evolutionary rates (substitution per site per year) were estimated and used as priors in the present study: 1.51 × 10^−3^ ± 2.66 × 10^−5^, 2.78 × 10^−3^ ± 2.30 × 10^−6^, 5.20 × 10^−3^ ± 6.85 × 10^−5^, and 3.71 × 10^−3^ ± 9.00 × 10^−5^ for the subtypes 1b, 2a, 3b, 6a Core-E1 sequences, respectively; 1.07 × 10–3 ± 2.48 × 10–5 and 4.28 × 10–3 ± 1.71 × 10–5 for the subtypes 1b, 2a NS5B sequences, respectively. After generating the XML files and importing them into BEAST, the Markov Chain Monte Carlo (MCMC) chain was run for 300,000,000 steps and sampled every 10,000 steps, ensuring that sufficient sampling has been achieved, indicated by the estimated effective sampling sizes (ESSs) greater than 200. After that, the program TreeAnnotator was used to generate the maximum clade credibility (MCC) tree as well as to summarize the posterior density of trees to calculate the posterior probabilities for the ancestral geographic states. Moreover, Tracer was used to explore the output of BEAST and the FigTree program was applied to display the resulting posterior trees.

### Statistical analysis

SPSS 16.0 was used to analyze the data. Univariate analysis was performed with Fisher’s exact test for each risk factor. A *p* value of less than 0.05 was considered statistically significant.

### Sequence Accession Numbers

The obtained sequences were submitted to GenBank and given the following accession numbers: KM523551-KM523588, KM523593-KM523599, KP993301-KP993373, and KP993374-KP993464.

## Results

### Prevalence of HCV infection

A total of 570 patients were tested positive for anti-HCV at the Department of Clinical Laboratory of Tongji Hospital during the study period and thus enrolled in this study. There were 274 men and 296 women, with ages ranging from 18 to 70 years old (mean: 38.7 ± 12.1 years old). Of these patients, 252 subjects were HCV RNA positive as determined by a real-time PCR system, giving a prevalence of 44.2%. We speculated that the low prevalence of HCV RNA among anti-HCV-positive patients could be due to the high false-positive rate of the used assay for anti-HCV (Architect reagents). With this method, false-positive results have been found in about 95% of the initial positive results with S/CO values less than 5 [25], which accounted for around one third (185/570) of the subjects recruited in this study, indicating a low prevalence of HCV RNA in the cohort.

### Genotype distribution, demography, and risk factors

**Fig 2:**
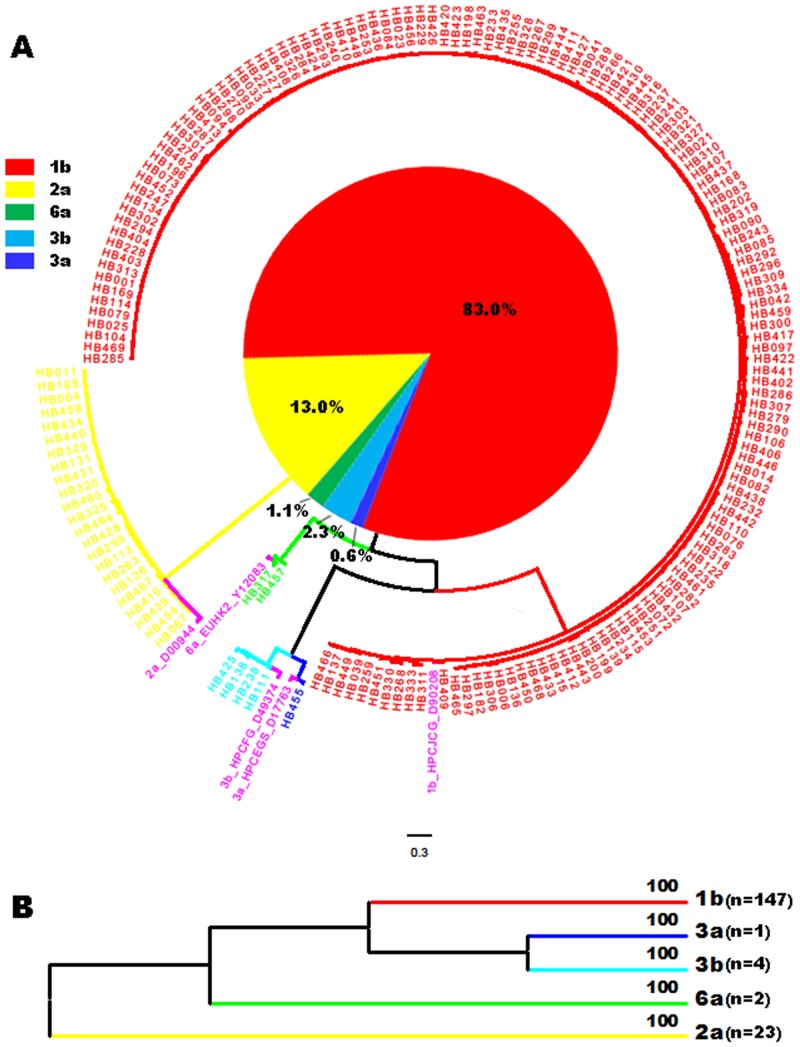
Circle form of a phylogenetic tree based on the NS5B sequences from 177 study subjects co-analyzed with 5 references for 5 assigned subtypes. Different subtypes are shown in different colors, as indicated on the left of the tree. All references are shown in magenta with their format as: subtype_isolate ID_GenBank accession number. The bar at the base of the figure shows the scale for nucleotide substitution per site. The pie chart inside the tree indicates the percentages of the different HCV subtypes of the 177 isolates obtained in this study. (B) Topology tree converted from the circular form of the tree shown in panel A. Each branch represents a single subtype and is labeled to the right with the number of isolates in parentheses and at the top with the level of bootstrap support.

Among the 252 HCV RNA-positive subjects determined by a real-time PCR system, both Core-E1 and NS5B were detected and sequenced in 164 patients while only the NS5B sequences were obtained and genotyped in 13. For the latter 13, the more conserved Core region was successfully amplified and genotyped. Eventually, consistent genotyping results were obtained for each sample assessed, indicating no mixed infection or viral recombination event in this cohort. Failure to amplify and sequence the viral genome in the remaining samples may be due to a long period of exposure at room temperature before storing at -70°C and/or low viral loads. Fig 2 shows the phylogenetic tree of the Hubei sequences and standard reference sequences. Table 1 shows the distribution of the different subtypes together with demography and related risk factors. The distribution of HCV subtypes can be summarized as follows: subtype 1b, 83.0% (147/177); 2a, 13.0% (23/177); 3b, 2.3% (4/177); 6a, 1.1% (2/177); 3a, 0.6% (1/177).

**Table 1:** Demographics and risk factors of patients associated with HCV subtypes. Data are n or n (%) unless otherwise stated. HBsAg: Hepatitis B surface antigen; anti-HIV: HIV-1 antibody; anti-TP: antibody to treponema pallidum.

	HCV subtype			
Factor	1b (n = 147)	2a (n = 23)	3b (n = 4)	*P* value
**Gender**				0.333
Male/female	109/128	13/10	3/1	
**Age (years)**				0.006
≤20	3 (2.0)	0 (0.0)	0 (0.0)	
21–30	12 (8.2)	2 (8.7)	0 (0.0)	
31–40	10 (6.8)	0 (0.0)	3 (75.0)	0.004
41–50	34 (23.1)	2 (8.7)	1 (25.0)	
>50	88 (59.9)	19 (82.6)	0 (0.0)	0.003
**Risk behavior** ^**a**^				0.000
Blood transfusion	79 (53.8)	12 (52.2)	0 (0.0)	0.000
Paid blood donation	44 (29.9)	2 (8.7)	0 (0.0)	
Hemodialysis	5 (3.4)	0 (0.0)	0 (0.0)	
Intravenous drug use	0 (0.0)	0 (0.0)	4 (100.0)	0.000
Sexual contact	4 (2.7)	6 (26.1)	0 (0.0)	
Unknown^b^	15 (10.2)	3 (13.0)	0 (0.0)	
**Education level**				0.004
>High school	36 (24.5)	0 (0.0)	0 (0.0)	
Middle or high school	80 (54.4)	13 (56.5)	4 (100.0)	
≤Primary school	31 (21.1)	10 (43.5)	0 (0.0)	
**Job**				0.035
Worker	55 (37.4)	8 (34.8)	0 (0.0)	
Peasant	48 (32.7)	10 (43.5)	0 (0.0)	
Civil servant	12 (8.2)	1 (4.3)	0 (0.0)	
Student	3 (2.0)	0 (0.0)	0 (0.0)	
Merchant	13 (8.9)	0 (0.0)	3 (75.0)	0.001
Unemployed	16 (10.8)	4 (17.4)	1 (25.0)	
**HBsAg status**				0.256
Positive/negative	15/132	4/19	1/3	
**Anti-HIV status**				1.000
Positive/negative	1/146	0/23	0/4	
**Anti-TP status**				0.161
Positive/negative	5/142	0/23	1/3	

^a^HCV subtypes 1b, 2a, and 3b were strongly associated with risk behaviors (Fisher’s exact test, *P* < 0.05).

^b^Unknown, patients where the source of infection was unclear and unknown.

Regarding HCV subtype-associated risk factors, there were no significant differences among individuals with various subtypes in gender as well as HBsAg, anti-HIV, and anti-TP status. The linked risk factors for HCV infections differed significantly, with blood transfusion and paid blood donation before 1997 being the main risk factors for subtype 1b infection, while subtype 2a was found not only in patients with blood transfusion and paid blood donation before 1997 but also in individuals with high risk sexual behaviors (e.g., multiple sexual partnership and male-to-male sex). The four subtype 3b isolates, along with one 3a and two 6a isolates, were only found in IDUs. As for age, patients with subtype 1b covered a wide range of ages. However, those with 2a were found mainly in two age groups, 21–30 and older than 40, with the former age group exclusively being MSM (men who have sex with men) and the latter mostly being those once received transfusion or sold blood before 1997. Those with 3b were aged from 31 to 50 years. Patients with 1b received education levels ranging from illiterate to college, while all the patients with 2a and 3b received education levels not above high school. With respect to occupation, subjects with subtype 1b had a variety of jobs, while those with 2a were mainly workers and peasants, who are less well-off in China at present. The four subtype 3b subjects included three merchants and one unemployed person, who are better off or have abundant time (Table 1).

### Phylogeographic analysis

To explore the origins and possible migration patterns of HCV in Hubei Province, phylogeographic trees were reconstructed using the Bayesian phylogeographic inference framework implemented in the BEAST software, which were based on a large number of reference sequences and Hubei sequences. The former included those from 16 provinces and municipalities of China [5], obtained in a nationwide study, as well as Vietnam [24]. The latter included sequences obtained in this study as well as sequences from blood donors [5], HCV and HIV co-infected patients [14], and IDUs [13] in Hubei Province. Hence, our findings are more likely to represent the true situation of HCV infection in this area. Briefly, four trees were generated, each representing one of the four common subtypes in Hubei, i.e., 1b, 2a, 3b, and 6a.

**Fig 3:**
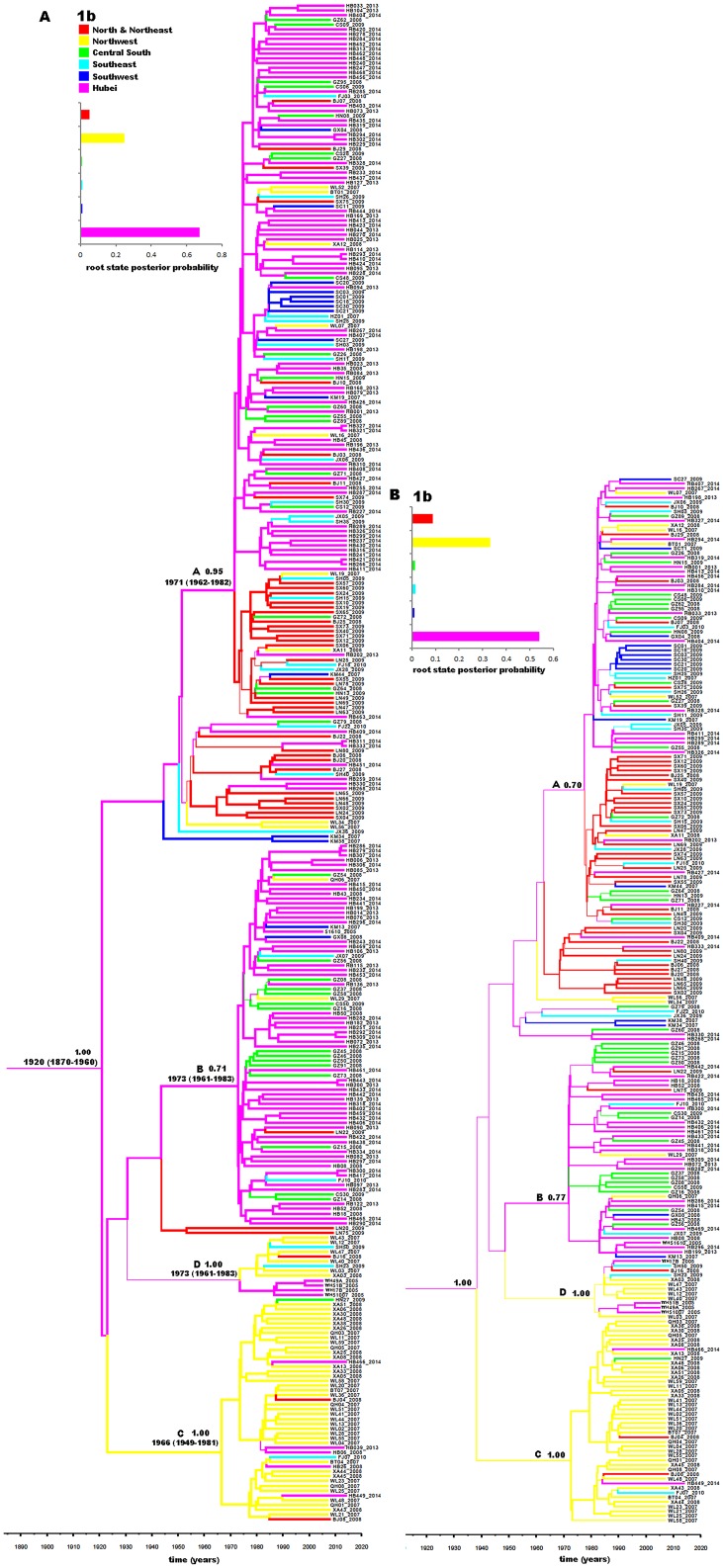
Phylogeographic tree reconstructed with the partial Core-E1 sequences of subtype 1b isolates. (A) A total of 333 subtype 1b sequences were used to generate the phylogeographic tree: 147 obtained in this study, indicated by HB and three digits (e.g., HB001), 5 from intravenous drug users (IDUs) in Wuhan city of Hubei Province, indicated by WH and several digits and/or characters [13], and 180 from blood donors from the 17 provinces and municipalities in China, including 15 from Hubei Province, which were named by HB and two digits (e.g., HB01) [5]. Branches are colored according to the most probable location state of their descendent nodes. Branch width is proportional to the probability value of the inferred ancestral geographical state. To the upper left of the tree are its root location state posterior probability distribution and the color scheme for different geographic origins. At the respective nodes, posterior probabilities of >0.70 are shown along with the estimated time points of the origin for the nodes. Below the tree is a time scale, which indicates the time of HCV origin and evolution. The four intra-genotypic lineages discussed in the main text are labeled A, B, C, and D. (B) The same phylogeographic tree was generated on the basis of the aforementioned dataset in panel A except that only 49 Hubei sequences were included, which were selected according to a scenario that no more than 3 sequences were retained from each phylogenetic cluster of the phylogeographic tree in panel A.

Fig 3A presents a phylogeographic tree estimated on the basis of 372 subtype 1b sequences. Altogether, four groups (Groups A, B, C, and D) were identified, showing significant posterior probabilities of 0.95, 0.71, 1.00, and 1.00, respectively. Between Groups A and B, There appeared two additional groups, one of which contained sequences mostly from Hubei and the north-northeast. However, they showed no significant posterior probabilities. Group A sequences showed a wide range of geographic distribution and the sequences from different regions were cross distributed, which suggested a simultaneous dissemination nationwide. However, these isolates may have descended from a common ancestor in Hubei dated around 1971 (95% CI: 1962–1982). Sequences in Group B were mainly from Hubei and the central south, in particular, Guangdong Province, indicating that subtype 1b was locally epidemic in these two regions. It seems that this group of 1b strains may have originated from a Hubei ancestor dated around 1973 (95% CI: 1961–1983). In contrast to Groups A and B, which contained sequences with a mixture of geographic origins, Groups C and D contained sequences mainly from a single region, the northwest. Both of them may also have descended from Hubei ancestors, dated around 1973 (95% CI: 1960–1984) and 1981 (95% CI: 1972–1990), respectively. It should be mentioned that Group D isolates were mainly from the IDUs in the northwest and Hubei Province, which suggests a common IDU network between these two regions. To further confirm the aforementioned findings and to avoid potential influences of a heavy weight of Hubei sequences on evolutionary analysis, an additional phylogeographic tree was reconstructed (Fig 1B) based on the same dataset except that only 49 Hubei sequences were included. These Hubei sequences were selected according to a scenario that no more than 3 sequences were retained from each phylogenetic cluster. As a whole, there appeared a trend of 1b migration from Hubei to other parts of China. Among them, the north-northeast and northwest became the second source regions to disseminate 1b trains to other parts of China. These findings were further demonstrated by the phylogeographic trees estimated on the relatively short NS5B sequences (S1 Fig).

**Fig 4:**
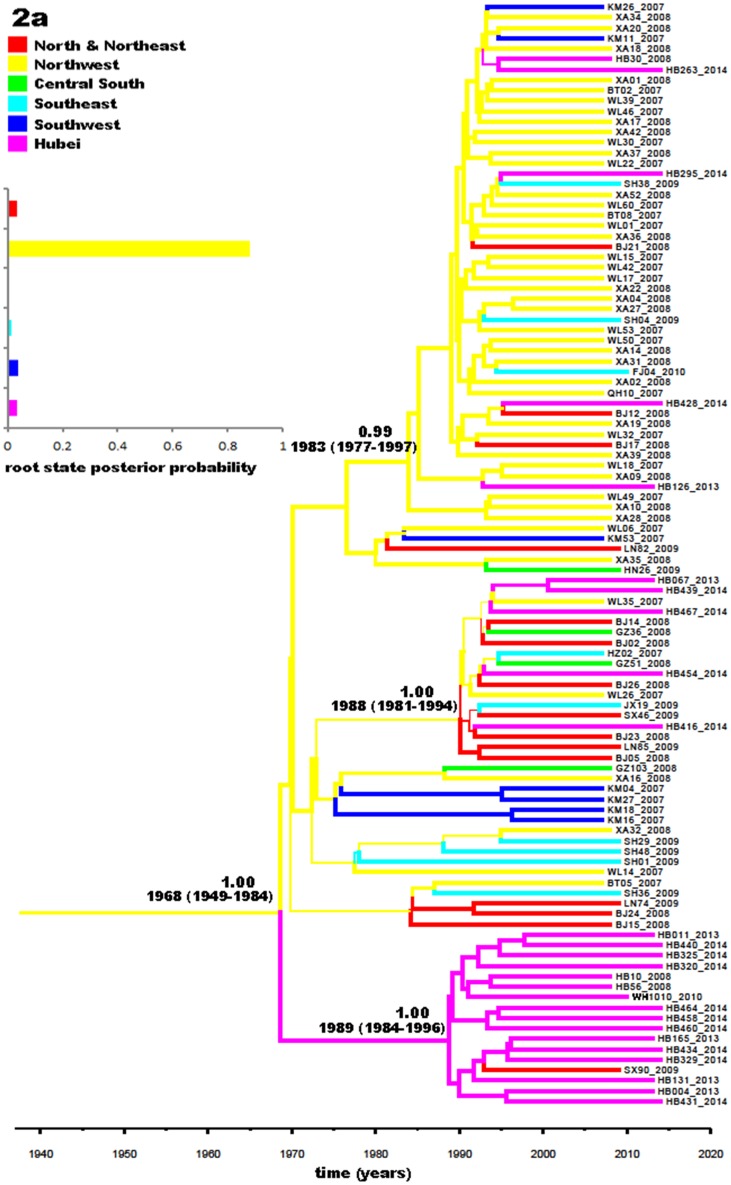
Phylogeographic tree generated with the partial Core-E1 sequences of subtype 2a isolates. A total of 106 subtype 2a sequences were used to generate the phylogeographic tree: 23 obtained in our study, 1 from a male homosexual in Wuhan City [14], and 81 from blood donors from the 17 provinces and municipalities in China, including 2 from Hubei [5]. The indications in this legend are the same as those in Fig 3.

Fig 4 presents a phylogeographic tree for 106 subtype 2a sequences. Overall, these 2a sequences can be classified into three big groups, showing significant posterior probabilities of 0.99, 1.00, and 1.00, respectively. Among them, four loose groups were observed, but they showed no significant posterior probabilities. The upper group had the largest number size and contained sequences mainly from the northwest. The middle one had the majority of its sequences originating in Hubei, while the lower one displayed branches with sequences with a mixture of geographic origins. It seems that 2a in China may have descended from a common ancestor in the northwest dated around 1968 (95% CI: 1949–1984), from where it was transmitted to other regions and became locally epidemic in Hubei and the northwest. The same geographic distribution pattern and migration trend of 2a strains were also observed in the phylogeographic tree estimated with the NS5B sequences (S2 Fig), except that the upper group showed a posterior probability of 0.61.

**Fig 5:**
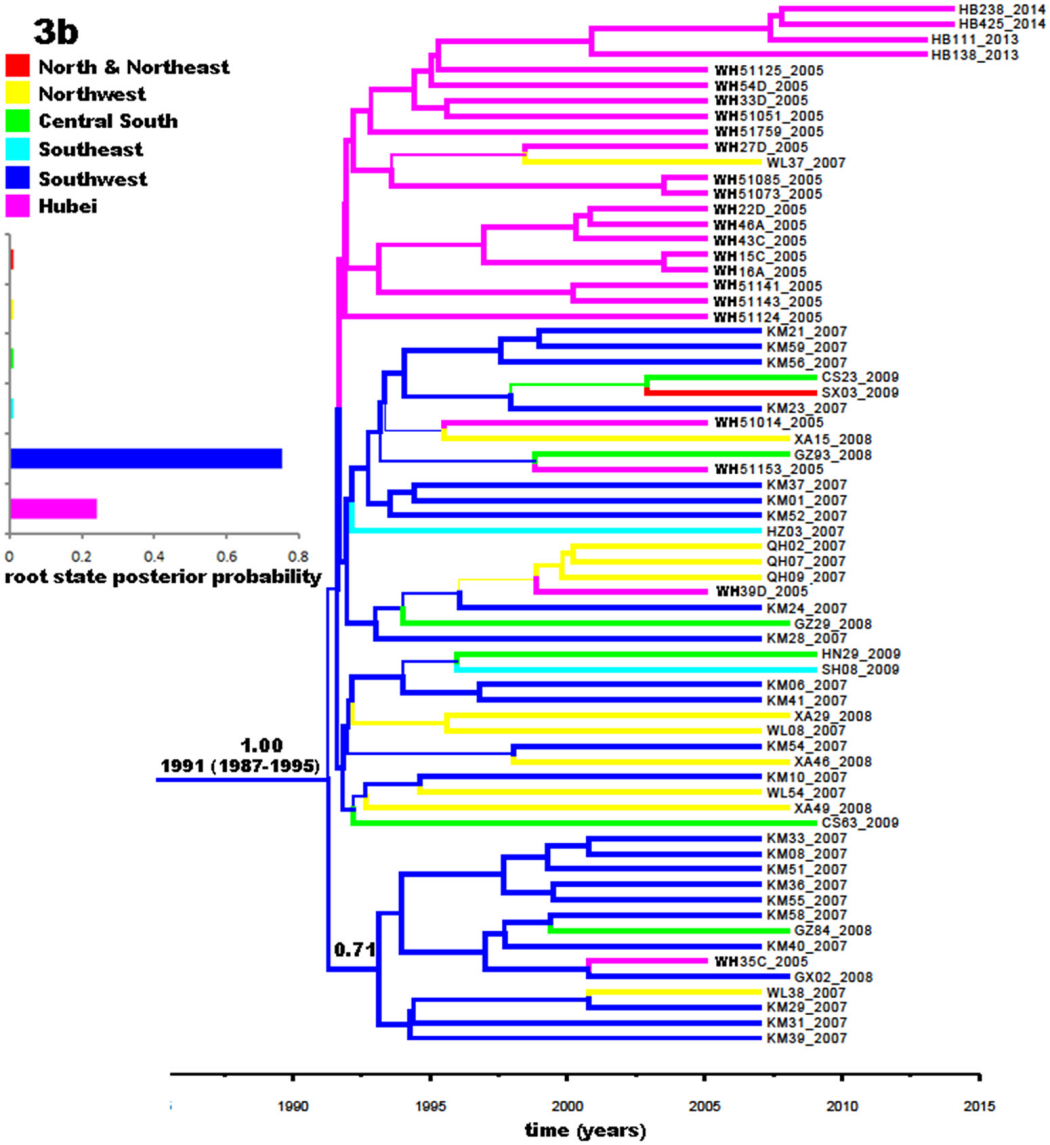
Phylogeographic tree generated with the partial Core-E1 sequences of subtype 3b isolates. A total of 68 subtype 3b sequences were used to estimate the phylogeographic tree: 4 obtained in this study, 20 from IDUs in Wuhan City [14], and 44 from blood donors from the 17 provinces and municipalities in China [5]. The indications here are the same as those in Fig 3.

Fig 5 displays a phylogeographic tree for 68 subtype 3b sequences. As a whole, four groups were visible, but only the lowest one showed significant posterior probability of 0.71 and this group featured sequences mainly from the southwest, in particular, Yunnan Province. The upper group contained sequences mostly from Hubei except for one from the northwest, suggesting a local epidemic in this area. The two middle groups showed substantial geographic interspersion. As regards migration routes, 3b in China may have originated from Yunnan in the southwest, whereupon spread to other regions.

**Fig 6:**
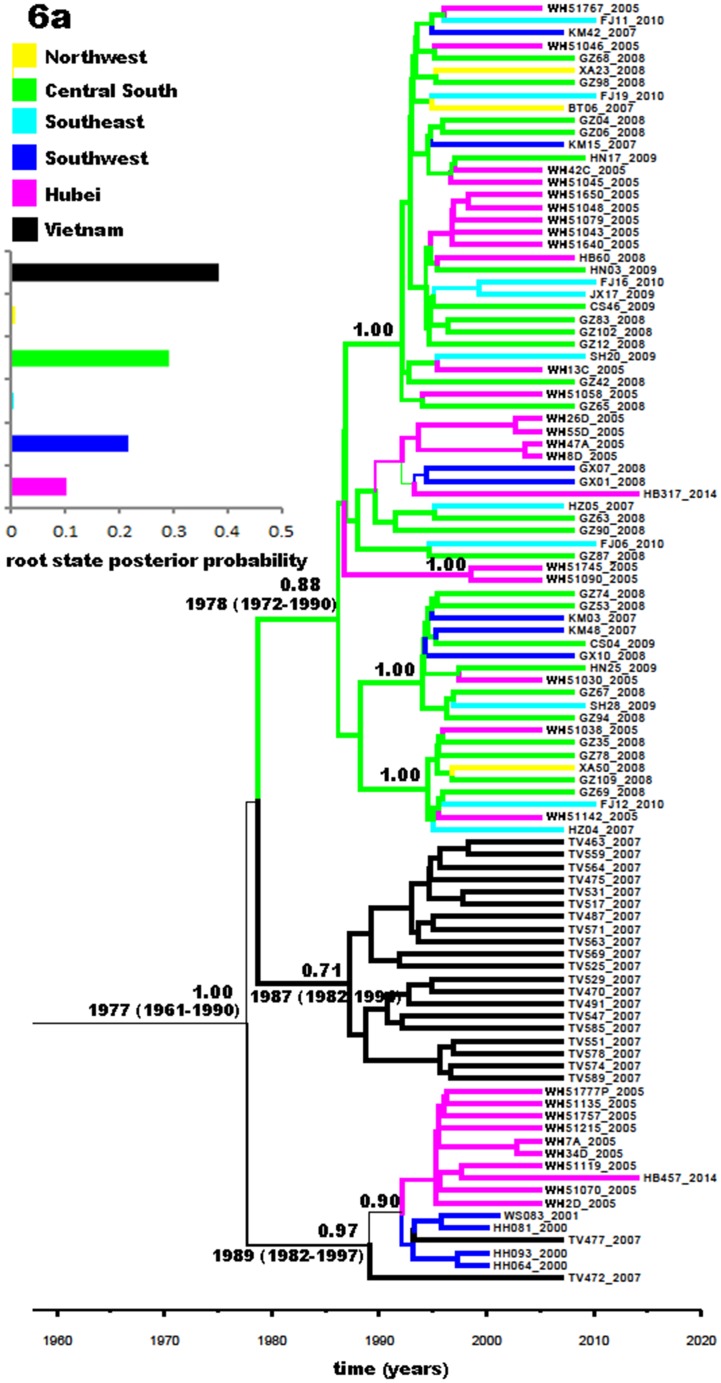
Phylogeographic tree generated with the partial Core-E1 sequences of subtype 6a isolates. A total of 103 subtype 2a sequences were used to reconstruct the phylogeographic tree: 2 obtained in this study, 29 from IDUs in Wuhan City [14], 46 from blood donors from the 17 provinces and municipalities in China, including 1 Hubei sequence [5], 4 from IDUs in Yunnan Province, and 22 from Vietnamese IDUs. The indications here are the same as those in Fig 3.

Fig 6 presents a phylogeographic tree reconstructed on the basis of the 103 Core-E1 sequences of subtype 6a. Overall, three subsets were visible, showing posterior probabilities of 0.88, 0.71, and 0.97, respectively. The upper subset featured sequences mainly from the central south and can be further divided into five groups. Among them four displayed full posterior probabilities of 1.00. The middle subset contained sequences exclusively from Vietnam and showed a posterior probability of 0.71. The lower one showed a posterior probability of 0.97 and contained sequences from three areas: Hubei, Yunnan, and Vietnam,. As a whole, there appeared a trend of 6a migration in China: descended from Vietnam, 6a was first introduced into Yunnan and Guangdong, and then from Guangdong to other regions. The Hubei strains seemed to have two ancestors, which were located in Guangdong and Yunnan, respectively. Since its introduction, 6a may have become local epidemics in Hubei, indicated by four clusters of Hubei sequences observed in the phylogeographic tree.

## Discussion

In this study, 1b (83.0%) and 2a (13.0%) were the two most common subtypes in patients who were tested for HCV prior to transfusion, followed in frequency by 3b (2.3%), 6a (1.1%), and 3a (0.6%). The genotypic distribution of HCV among these Hubei patients is consistent with that in the overall population of China, in which the main circulating HCV subtype is 1b, followed by 2a [5, 11]. However, another study by Zhao et al. [14], which was performed on patients with HIV/HCV co-infection in Wuhan, the capital of Hubei Province, reported that 6a was the most prevalent subtype in this population, accounting for 39.3% of these patients, followed by 1b (24.7%), 3b (18.0%), 3a (9.8%), 1a (4.9%), and 2a (3.3%). The discrepancy between our results and those by Zhao et al. could be due to the different modes of HCV acquisition. In Zhao’s study, intravenous drug use (IDU) was the predominant risk factor for HCV infection (87.3%), which has been strongly associated with subtype 6a in China [26]. However, in our study, the most common risk factors were paid blood donation and transfusion, which have been demonstrated to be the main risk factors for 1b infection.

Based on samples from 411 volunteer blood donors from 17 provinces and municipalities, a recent survey on HCV migration patterns demonstrated that the five major subtypes (1b, 2a, 3a, 3b, and 6a) prevalent in China displayed distinct geographic distribution patterns [5], which has shed light on our understanding of HCV molecular epidemiology. However, in that investigation only 13 Hubei isolates were included, which cannot represent the true situation of HCV infection in this area as well as its relationship with other regions. In the present study, based on the above-mentioned dataset as well as a large number of additional sequences from Hubei, Yunnan, and Vietnam, four phylogeographic trees were reconstructed using the Bayesian phylogeographic inference framework implemented in BEAST, each representing one of the four subtypes, 1b, 2a, 3b, and 6a. The tree for 1b shows four major groups, which is consistent with the finding in the nationwide survey [5], where lots of sequences were utilized to construct the phylogeographic trees in this study. However, with the addition of a large number of Hubei sequences, the phylogeographic tree showed a more clear migration trend: 1b strains in China may have descended from Hubei Province, whereupon they were transmitted to other regions during the 1960s and 1970s. Among them, the north-northeast and northwest became the second source regions to disseminate 1b trains to other regions. We reasoned this could be due to two facts. First, in the 1950s, a cooperative medical system was developed in China, which provided basic care and preventive services (e.g., vaccination) to all people [27]. However, the limited medical knowledge and professional training received by the health-care providers, especially during the years 1966–1976 when the “Cultural Revolution” occurred and medical schools and specialist hospital departments were closed [27, 28], may have facilitated the dissemination of bloodborne viruses (such as HCV) by unsafe injection as well as traditional Chinese acupuncture [29, 30]. Second, located in central China and known as “the thoroughfare leading to nine provinces”, Hubei has been acting as an important national transportation hub, which made it a possible origin of 1b transmission in China at that time. This speculation however needs to be further investigated since the direct ancestor from Hubei Province was not found.

HCV subtype 2a is the second most predominant subtype in China, resulting in a nationwide prevalence of 14% [31–33]. In the nationwide survey [5], 2a strains in China were roughly classified into two major groups: one, being more monophyletic, predominates in the northwest and may, because of neighborhood, have been transmitted from Afghanistan through a drug trafficking route [34, 35]. The other one is disseminated nationwide at a lower density and may originate from the former commercial plasma donors during the 1990s [5]. In this study, 2a was also demonstrated to be the second major HCV subtype prevalent in Hubei. In addition to the two major groups observed in the nationwide investigation, another geographic lineage from Hubei was also observed, which suggests a local epidemic in this area. Furthermore, our results revealed that all the three groups of 2a strains in China may have their origins in the northwest. Since sequences from the sex- and transfusion-related HCV patients in Hubei were interspersed among the three groups of 2a sequences, we can therefore speculate that 2a may have been transmitted to the northwest of China from its neighbor, Afghanistan, via a drug trafficking route [34, 35]. After that, it evolved into three lineages, one epidemic in the northwest, one prevalent in Hubei in the central, and the third one disseminating nationwide. All of them may have been transmitted through unsafe blood transfusion and/or sexual contact.

In the present study, all the 3b and 6a strains obtained in Hubei were exclusively from IDUs, which is in line with previous findings that 3b and 6a were detected more often among IDUs than among other people [8, 10, 26]. The phylogeographic tree constructed in this study suggested a trend of subtype 3b migration from the southwest to the northwest and Hubei, and sporadically to other regions. The migration trend for 3b inferred in this study is consistent with the finding that 3b originated in Southeast Asian countries (such as Vietnam) and was introduced to China via IDU through Yunnan Province, which borders the Golden Triangle in Southeast Asian countries [36]. For 6a strains, there appeared a migration trend from Vietnam first to Yunnan and Guangdong, and then from Guangdong to other regions. The migration pattern for 6a inferred in our study is somehow different from that by Fu et al [24], who speculated that 6a in China originated in Vietnam, from where it was first introduced to Yunnan and Guangxi, and then form Guangxi to Guangdong, where 6a became locally epidemic and then spread to other regions [24]. The possible reasons for this discrepancy may be that the sample size of Guangxi isolates was relatively small, and they were obtained in a recent study and therefore can only represent recent cases.

In this study, all the subjects were recruited from the biggest hospital in Hubei Province and were distributed in various departments. Characterized by chronicity and latency, HCV infections are usually found when patients receive tests for a panel of pathogens prior to transfusion and are determined positive for anti-HCV. In addition, subjects enrolled in our study came from the whole province and covered a wide range of ages and occupations. Our investigation can therefore represent the true situation of HCV infection in this area.

In conclusion, our study suggests that there are five HCV subtypes, 1b, 2a, 3a 3b, and 6a, present in Hubei Province, with subtype 1b most prominent, followed by 2a and 3b. Among them, the 1b strains in Hubei Province may have served as the origins of this subtype in China, while other subtypes in Hubei may have a variety of geographic origins. Our study also demonstrated that most of the HCV infection cases at the moment can be attributed to the contaminated blood donation in the 1990s. However, such epidemiological pattern is constantly evolving as HCV acquisition modes change. In the younger population, post-transfusion infection has seldom occurred since the implementation of mandatory HCV screening of blood and blood products in the early 1990s [6, 31]; while the number of IDU-associated cases has increased dramatically in accordance with the increasing number of IDUs in recent years [3, 8, 10, 11, 14, 36]. In addition, sexual contact is also becoming a common transmission route for both HIV and HCV in Hubei [14]. Therefore, measures should be taken to interfere with the possible transmission routes, thereby preventing HCV transmission effectively.

## Supporting Information

S1 Fig Phylogeographic tree reconstructed with the partial NS5B sequences of subtype 1b isolates. (A) A total of 324 subtype 1b sequences were used to generate the phylogeographic tree: 138 obtained in this study, 6 from HCV and HIV co-infected patients from Wuhan City of Hubei Province, and 180 from blood donors from the 17 provinces and municipalities in China, including 15 from Hubei Province. (B) The same phylogeographic tree was generated on the basis of the aforementioned dataset in panel A except that only 45 sequences from Hubei Province were included, which were selected according to a scenario that no more than 3 sequences were retained from each phylogenetic cluster of the phylogeographic tree in panel A. All other indications are the same as described in Fig 3.
(TIF)

S2 Fig Phylogeographic tree generated with the partial NS5B sequences of subtype 2a isolates. A total of 104 subtype 2a sequences were used to generate the phylogeographic tree: 23 obtained in our study and 81 from blood donors from the 17 provinces and municipalities in China, including 2 from Hubei [5]. The indications in this legend are the same as those in Fig 3.
(TIF)
