# Supplementary figures and images for: Genotype Distribution and Molecular Epidemiology of Hepatitis C Virus in Hubei, Central China
Source: PLoS One. 2015 Sep 1;10(9):e0137059. doi: 10.1371/journal.pone.0137059 (PMC4556612; doi:10.1371/journal.pone.0137059)

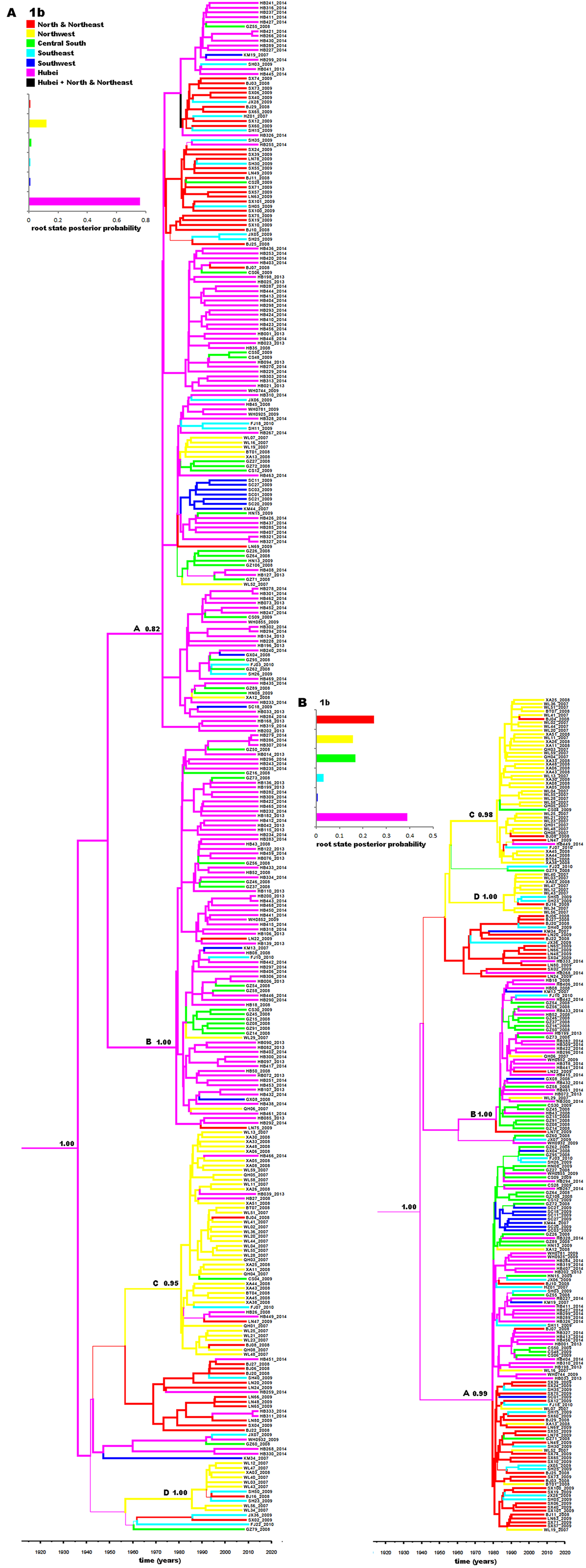

Supplement: S1 Fig — (A) A total of 324 subtype 1b sequences were used to generate the phylogeographic tree: 138 obtained in this study, 6 from HCV and HIV co-infected patients from Wuhan City of Hubei Province, and 180 from blood donors from the 17 provinces and municipalities in China, including 15 from Hubei Province. (B) The same phylogeographic tree was generated on the basis of the aforementioned dataset in panel A except that only 45 sequences from Hubei Province were included, which were selected according to a scenario that no more than 3 sequences were retained from each phylogenetic cluster of the phylogeographic tree in panel A. All other indications are the same as described in Fig 3. (TIF) [file pone.0137059.s001.tif]

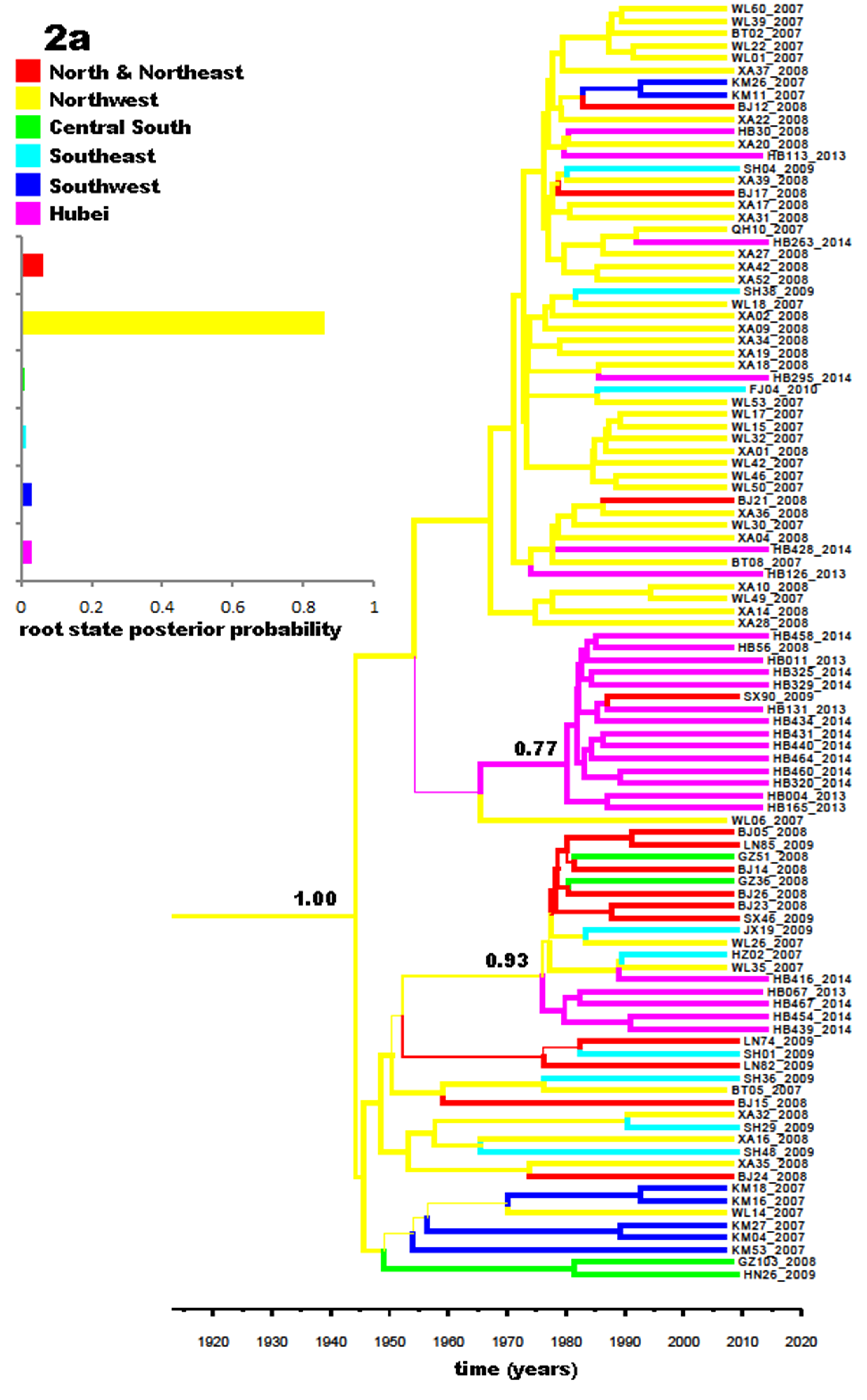

Supplement: S2 Fig — A total of 104 subtype 2a sequences were used to generate the phylogeographic tree: 23 obtained in our study and 81 from blood donors from the 17 provinces and municipalities in China, including 2 from Hubei [5]. The indications in this legend are the same as those in Fig 3. (TIF) [file pone.0137059.s002.tif]
